# Integrating neural networks with advanced optimization techniques for accurate kidney disease diagnosis

**DOI:** 10.1038/s41598-024-71410-6

**Published:** 2024-09-18

**Authors:** Samar Elbedwehy, Esraa Hassan, Abeer Saber, Rady Elmonier

**Affiliations:** 1 Department of Data Science, Faculty of Artificial Intelligence, Kafrelsheikh University, Kafrelsheikh, 33511 Egypt; 2 Faculty of Artificial Intelligence, Kafrelsheikh University, Kafrelsheikh, 33511 Egypt; 3 Department of Information Technology, Faculty of Computers and Artificial Intelligence, Damietta University, Damietta, 34517 Egypt; 4 Department of Internal Medicine, Faculty of Medicine, Al-Azhar University, New Damietta, Egypt

**Keywords:** Feature concatenation, Image classification, Optimizer, Vision transformer, Kidney diseases, Radiography

## Abstract

Kidney diseases pose a significant global health challenge, requiring precise diagnostic tools to improve patient outcomes. This study addresses this need by investigating three main categories of renal diseases: kidney stones, cysts, and tumors. Utilizing a comprehensive dataset of 12,446 CT whole abdomen and urogram images, this study developed an advanced AI-driven diagnostic system specifically tailored for kidney disease classification. The innovative approach of this study combines the strengths of traditional convolutional neural network architecture (AlexNet) with modern advancements in ConvNeXt architectures. By integrating AlexNet’s robust feature extraction capabilities with ConvNeXt’s advanced attention mechanisms, the paper achieved an exceptional classification accuracy of 99.85%. A key advancement in this study’s methodology lies in the strategic amalgamation of features from both networks. This paper concatenated hierarchical spatial information and incorporated self-attention mechanisms to enhance classification performance. Furthermore, the study introduced a custom optimization technique inspired by the Adam optimizer, which dynamically adjusts the step size based on gradient norms. This tailored optimizer facilitated faster convergence and more effective weight updates, imporving model performance. The model of this study demonstrated outstanding performance across various metrics, with an average precision of 99.89%, recall of 99.95%, and specificity of 99.83%. These results highlight the efficacy of the hybrid architecture and optimization strategy in accurately diagnosing kidney diseases. Additionally, the methodology of this paper emphasizes interpretability and explainability, which are crucial for the clinical deployment of deep learning models.

## Introduction

Kidney diseases have emerged as a significant global health concern, with chronic kidney disease affecting over 10% of the world's population. This condition, predicted to rise to the fifth leading cause of death by 2040, underscores the pressing need for effective control measures. Among the prevalent kidney ailments impeding normal renal function, kidney cysts, nephrolithiasis (kidney stones), and renal cell carcinoma (kidney tumor) pose substantial threats^1^. Kidney cysts, fluid-filled pockets on the kidney's surface, and nephrolithiasis, involving crystal concretion formation, impact approximately 12% of the global population. Renal cell carcinoma is identified as one of the top ten most common cancers worldwide. There are different types of data that researchers handled it; Text and images. Text datasets Also often contain valuable information derived from medical records, pathology reports, and patient histories, which can be leveraged to train machine learning models^2–5^.

Also, diagnostic tools such as X-ray, computed tomography (CT), B-ultrasound, and magnetic resonance imaging (MRI) play crucial roles in conjunction with pathology tests for accurate kidney disease diagnosis. CT scans, particularly valuable for their three-dimensional insights and detailed slice-by-slice imaging, offer a comprehensive understanding of kidney anatomy^6^.

Recognizing the urgency of addressing these challenges, the advancement of deep learning in vision tasks presents a compelling opportunity. Building artificial intelligence (AI) models capable of efficiently detecting kidney radiological findings has become imperative to assist medical professionals and alleviate the suffering of individuals affected by kidney diseases. While some studies have explored this domain, the scarcity of publicly available datasets remains a hindrance. Furthermore, past research has often relied on traditional machine learning algorithms, focusing on the classification of single disease classes, such as cysts, tumors, or stones, and occasionally utilizing ultrasound images. In light of these considerations, there is a growing need to expand the scope of AI applications, leveraging deep learning advancements for a more comprehensive approach to kidney disease detection.

### Feature concatenation

Feature concatenation plays a crucial role in enhancing the effectiveness of deep learning models, especially in tasks such as image classification. By combining different types of features extracted from diverse sources, feature concatenation enables the creation of a more comprehensive and informative representation of the input data. This process allows the model to leverage complementary information embedded in various aspects of the data, such as color, texture, or spatial features. Unlike traditional single-feature approaches, feature concatenation enables the model to capture a richer set of characteristics, potentially improving its ability to generalize and make accurate predictions. Moreover, this technique facilitates the integration of information from different modalities or feature extraction methods, leading to a more robust and nuanced representation. In essence, feature concatenation serves as a powerful tool for refining the input representation, contributing to the model's overall performance and its capacity to handle complex patterns and relationships within the data.

### The main contributions of this study are as follows

- Novel classification method: the paper proposes a new approach for classifying kidney diseases that demonstrates robust performance across various datasets, emphasizing the importance of interpretability and explainability for clinical applications.
- Advanced integration of neural networks: this study integrates features from AlexNet and ConvNexT to create a comprehensive and informative feature representation. This fusion leverages the strengths of both architectures, resulting in superior performance compared to individual models.
- Enhanced model performance: By combining AlexNet and ViT, the paper achieved improved discriminative ability, capturing a broader range of visual features and surpassing the performance of the individual models.
- Optimized training process: this study introduced a custom optimization technique based on Adam that dynamically adjusts the step size according to the gradient norm, leading to more efficient convergence in training the merged AlexNet and ConvNexT models.

The rest of the paper is organized as follows; in the next section; literature reviews. In Sect. "Motivation", the motivation. In Sect. "Proposed methodology", the proposed methodology is used in this paper, followed by Sect. “Experiments and results”. Finally, in Sect. "Conclusions"; the paper is concluded with future work.

## Literature reviews

The classification of kidney diseases is a pivotal area of research that holds significant implications for clinical diagnosis, treatment planning, and patient management. As the understanding of renal disorders continues to evolve, there has been a growing body of literature dedicated to exploring various methodologies and techniques for accurate and efficient kidney disease classification. This literature review seeks to provide a comprehensive overview of the existing research landscape, delving into the diverse approaches employed in the classification of kidney conditions. From traditional methods to the latest advancements in machine learning and deep learning, this review aims to distill key insights and trends, shedding light on the progress made in enhancing diagnostic accuracy and paving the way for more effective therapeutic interventions. Through a systematic exploration of relevant studies, this literature review endeavors to offer a synthesis of knowledge that not only underscores the current state of kidney disease classification but also identifies potential avenues for future research and technological innovation in this critical domain. Parakh et al.^7^ proposed the initial convolutional neural network (CNN) was responsible for delineating the urinary tract's extent, while the second CNN focused on identifying the presence of stones. The authors created nine model variations by combining different training data sources (S1, S2, or both, denoted as SB) with pre-trained CNNs using ImageNet and GrayNet, as well as without pretraining (Random). The accuracy of GrayNet-SB, at 95%, surpassed that of ImageNet-SB (91%) and Random-SB (88%).

The research of Kuo et al.^8^ aims to enhance the prediction of kidney function and chronic kidney disease (CKD) through kidney ultrasound imaging, develop a model integrating the ResNet architecture, pre-trained on the ImageNet dataset, to estimate the glomerular filtration rate (eGFR) and CKD status from 4505 labeled kidney ultrasound images. The model demonstrated a strong correlation (Pearson coefficient of 0.741) between AI-based and creatinine-based GFR estimations and achieved 85.6% accuracy in classifying CKD status, outperforming experienced nephrologists (60.3%–80.1%).

Sudharson et al.^9^ utilized an ensemble technique, amalgamating diverse pre-trained Deep Neural Networks (DNNs) such as ResNet-101, ShuffleNet, and MobileNet-v2. The ultimate predictions were determined through the majority voting technique, resulting in a peak classification accuracy of 96.54% during testing with high-quality images and 95.58% during testing with noisy images.

Aksakallı et al.^10^ proposed the examination encompassed diverse machine learning approaches, including Decision Trees (DT), Random Forest (RF), Support Vector Machines (SVC), Multilayer Perceptron (MLP), K-Nearest Neighbor (kNN), Naive Bayes (BernoulliNB), and deep neural networks employing Convolutional Neural Network (CNN). The experimental outcomes revealed that the Decision Tree Classifier (DT) yielded the most favorable classification results. Specifically, this method attained the highest F1 score, achieving a success rate of 85.3% when employing the S + U sampling method.

Liu et al.^11^ focuse on making deep learning techniques more accessible for clinical users in the field of microscopic image classification by developing AIMIC, out-of-the-box software that requires no programming knowledge. AIMIC integrates advanced deep learning methods and data preprocessing techniques, allowing users to train new networks and infer unseen samples seamlessly. The platform was evaluated on four benchmark microscopy image datasets, demonstrating its effectiveness in selecting suitable algorithms for entry-level practitioners. Notably, the ResNeXt-50–32 × 4d model achieved the highest performance with an average accuracy of 96.83% and an average F1-score of 96.82%, making it the preferred choice for microscopic image classification. Additionally, MobileNet-V2 provided a good balance between accuracy (95.72%) and computational cost, with an inference time of 0.109 s per sample, making it a viable option for scenarios with limited computing resources.

Srivastava et al.^12^ used machine learning models (SVM, KNN, Random Forest, Decision Tree, AdaBoost) with the normalized dataset with an accuracy of 98.75%. Baygin et al.^13^ proposed a novel transfer learning-based image classification method called ExDark19. This method utilized iterative neighborhood component analysis (INCA) to select the most informative feature vectors, which were then input into a k nearest neighbor (kNN) classifier for kidney stone detection. Their results achieved an accuracy of 99.22% with a ten-fold cross-validation strategy and 99.71% using the hold-out validation method.

Nazmul Islam et al.^14^ employed a total of six machine learning models, with three being founded on advanced variants of Vision Transformers, namely EANet, CCT, and Swin Transformers. The remaining three models were based on deep learning architectures, ResNet, VGG16, and Inception v3, with adjustments made to their final layers. Despite commendable performances from the VGG16 and CCT models, the Swin Transformer emerged as the top performer in terms of accuracy, achieving an impressive accuracy rate of 99.30 percent. In this investigation, diverse physiological parameters were considered alongside the application of various machine learning (ML) techniques. Different ML models, including Support Vector Machines (SVM), K-Nearest Neighbors (KNN), Random Forest, Decision Tree, and AdaBoost, were trained using a normalized dataset, resulting in an impressive accuracy of 98.75%, perfect sensitivity (100%), high specificity of 96.55%, and a notable f1 score of 99.03%.

Subedi et al.^15^ explore the potential of a novel model called Vision Transformer (ViT), which was initially designed for natural language processing (NLP) tasks but shows promise for medical image classification. ViT’s capabilities are further enhanced by coupling it with Fully Connected Networks (FCN). This combination merges the feature extraction capabilities of ViT with the classification ability of FCN, ultimately overcoming the challenge of detecting kidney-related issues with greater accuracy and reliability with an accuracy of 99.64%.

Asif et al.^16^ introduced "StoneNet” which is based on MobileNet using depthwise separable convolution, offering a low-cost solution compared to existing models with drawbacks such as high computational costs and lengthy training times. Their model achieved accuracy at 97.98%, with short training and testing times of 996.88 s and 14.62 s, respectively. Qadir et al.^17^ focused on the Densenet-201 model for feature extraction with Random Forest being the chosen method. They achieved an accuracy rate of 99.719%. Table 1 presents the related work for the kidney classification.

**Table 1:** Related work in kidney classification using different datasets.

Reference	Year	Models	Dataset	Evaluation
Parakh et al.^7^	2019	Combined CNN	A specific dataset derived from a hospital's repository of CT scans	Accuracy = 95
Kuo et al.^8^	2019	ResNet	4505 high-quality images with annotations measuring kidney size, filtered from 1446 uniquely identifiable primary sonographic studies of 1299 patients	Accuracy = 85.6%
Sudharson et al.^9^	2020	Ensemble learning using ResNet-101,ShuffleNet, and MobileNet-v2	Collected dataset of 5490 images	Accuracy = 95.58
Aksakallı et al.^10^	2021	Convolutional Neural Network (CNN)	221 kidney x-ray images obtained from the Urology Department of Ataturk University	Accuracy = 83.5
Liu et al.^11^	2022	ResNeXt-50-324d is	C-NMC dataset	Accuracy = 96.83
Srivastava et al.^12^	2022	machine learning models (SVM, KNN, Random Forest, Decision Tree, AdaBoost)	The Indians Chronic Kidney Disease (CKD) dataset consists of 400 instances and 24 attributes with 2 classes	Accuracy = 98.75, **100%** Sensitivity, 96.55% Specificity
Baygin et al.^13^	2022	ExDark19 model	1799 CT images	Accuracy = 99.22%
Nazmul Islam et al.^14^	2022	Swin Transformer	12,446 CT images	Accuracy = 99.3, precision and recall reaching 99.15%, 99.15%, respectively with 0.99987 AUC
Subedi et al.^15^	2023	ViT + FCN	12,446 CT images	Accuracy = 99.64
Asif et al.^16^	2023	StoneNet	1799 CT images	Accuracy = 97.98%
Qadir et al.^17^	2023	Densenet-201 model	12,446 CT images	Accuracy = 99.719
Sasikaladevi et al.^18^	2024	HCNN	12,446 CT images	Accuracy = 99.71
This study	2024	Alex + ConvNexT	12,446 CT images	**Accuracy = 99.85**, precision, recall, and specificity reaching **99.89%, 99.95%, and 99.83%** respectively

Significant values are in [bold].

Sasikaladevi et al.^18^ address the critical need for early and automatic detection of chronic kidney disease (CKD) from radiology images using deep learning techniques. The dataset used contains 12,446 unique CT scan images. Deep features were extracted from these images, and hyperedges were generated to construct hypergraphs representing the renal images. These hypergraphs were then used in a hypergraph convolutional neural network for representational learning. The model was validated using a hold-out dataset, and deep learning metrics including precision, recall, accuracy, and F1 score were used to evaluate its performance. The proposed model demonstrated a superior validation accuracy of 99.71%, outperforming other state-of-the-art algorithms. This robust digital-twin model facilitates early diagnosis of kidney diseases and aids nephrologists in better prognosis of kidney-related abnormalities.

## Motivation

The urgent need to improve patient care and medical diagnostics in the field of renal health is the driving force behind the kidney classification paper. Kidney illnesses are a major global health concern, encompassing both acute and chronic ailments. Timely and accurate categorization of these ailments is essential for efficient treatment strategy development and patient supervision.

Several factors contribute to the motivation for kidney classification research:

- Clinical Importance: Diagnosing kidney disorders accurately can be challenging due to their wide range of etiologies and symptoms. Enhancing classification techniques helps medical professionals better comprehend various kidney disorders and customize treatment plans based on individual disease profiles.
- Early Identification and Intervention: It's critical to identify kidney disorders early to launch prompt interventions that can halt the disease's progression and enhance patient outcomes. Classification models can help detect kidney function issues early on, which can result in more proactive and focused medical interventions.
- Application of Advanced Technologies: The development of complex models for the classification of renal disease is made possible by advances in machine learning, deep learning, and image processing techniques. Making use of these technologies has the potential to completely transform how accurate and effective diagnostic procedures are.

## Proposed methodology

The paper discusses the impact of the concatenating features for enhancing the accuracy of kidney disease classification using the merging of Alex-Net^19^ with other models such as (ViT^20^, Swin^21^, and ConvNexT^22^) and also the impact with using the modified Adam optimizer “Custom-Adam” instead of the popular optimizer “Adam”.

The paper compared its performance with more recent architectures such as VGG and ResNet. The results show that the pre-trained VGG and ResNet models achieved accuracies of 91.73% and 94.63%, respectively. In contrast, more advanced models such as Vision Transformer (ViT), Swin Transformer, and ConvNexT achieved higher accuracies of 98.71%, 96.44%, and 96.44%, respectively. These findings highlight the superior performance of these newer architectures over Alex-Net. While Alex-Net has a well-established reputation in image classification tasks as its architecture is known for efficient feature extraction, which is crucial for accurately classifying kidney diseases from medical images.

Transformer models which include ViT and Swin have demonstrated remarkable performance in various computer vision tasks, particularly in capturing long-range dependencies and spatial relationships within images. For example, the main purpose for using the ViT model is self-attention mechanism allows it to capture global contextual information in images, enabling it to identify complex patterns and long-range relationships. But Swin optimizes the attention computation in Vision Transformers by limiting self-attention to non-overlapping local windows. This shifted window approach reduces the normally quadratic complexity of ViT to linear complexity concerning image size, making Swin more computationally efficient. Also, Swin is a hierarchical vision transformer that progressively merges adjacent patches as the network deepens. This hierarchical structure enables the model to manage features at various scales, enhancing the learning of robust and discriminative features compared to convolutional neural networks. But with ConvNexT model, incorporates modern techniques like hierarchical design and larger kernel sizes, enhancing its ability to handle diverse image features while maintaining the simplicity of traditional CNNs. The paper included these models to explore their potential to extract relevant features from medical images, which could contribute to improving diagnostic accuracy.

On the other side, changing the optimizer can significantly impact model accuracy, convergence speed, generalization ability, and overall stability. Therefore, choosing the right optimizer is crucial for optimizing machine learning models. The paper compared the effect of Adam^23^ and Custom_Adam optimizer on the dataset to find the Custom_Adam is better in most cases while the primary difference between the standard Adam optimizer and the Custom_Adam lies in the additional calculation and utilization of the gradient norm in the custom version. Specifically, Custom_Adam computes the norm of the gradient (denoted as norm_value) for each parameter $\theta$ with a non-None gradient:

1 $$norm\, value= \Vert {g}_{t}\Vert$$

This norm is then used in the custom update rule. The _update_rule method in Custom_Adams incorporates this norm_value along with the parameter $\theta$, gradient ${g}_{t}$​, and state during the update process, which can be expressed as:

2 $${\theta }_{t+1}= {\theta }_{t}-\frac{\alpha }{\widehat{{m}_{t}}}*\frac{\sqrt{\widehat{{v}_{t}}}}{norm\, value+\epsilon }$$

The parameter update in the standard Adam is as:

3 $${\theta }_{t+1}= {\theta }_{t}-\frac{\alpha }{\sqrt{{\widehat{v}}_{t}}+\in }*\widehat{{m}_{t}}$$

Additionally, Custom_Adam overrides the step method to include the gradient norm calculation and the call to the _update_rule, whereas the standard Adam optimizer utilizes its default step method without these extra computations. This enhancement allows Custom_Adam to adapt the learning rate based on the gradient's scale, potentially improving optimization performance. See the algorithm as the following .

**Figure:**
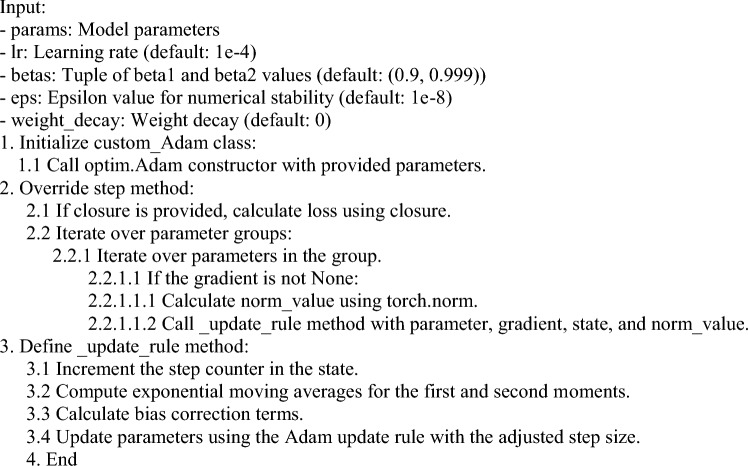
**Algorithm:** Custom_Adam

To accomplish this, the two actions listed can be taken: first; compare using four single vision models (ViT, Alex-Net, Swin, and ConvNexT) for extracting the features from images by using the optimizer Adam and the Custom_Adam. The second is to improve the extracting feature process using the concatenating features from the four vision models with the best optimizer that got from the first action; the vision models are (“Swin + ConvNexT”, “Alex-Net + ViT”, “Alex-Net + Swin” and “Alex-Net + ConvNexT” ). The paper finds as in Fig. 3 that concatenating the models Alex-Net with ConvNexT with Custom_Adam optimizer is the best value in accuracy 99.85% with metrics used for the evaluation such as average precision, recall, and specificity, reaching 99.89%, 99.95%, and 99.83% respectively.

### The methodology of this study for kidney classification involves several steps as in Fig. 1

**Fig. 1:**
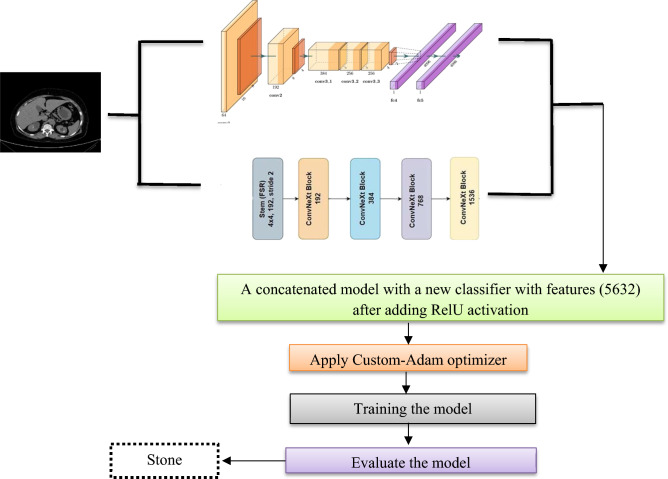
The methodology of this study for Kidney classification.

1. Image loading from the directory then applied using T.Compose to augment the training data, these transformations include random horizontal and vertical flips, random color jitter, resizing to 256 * 256 pixels, center cropping to 224 * 224 pixels, conversion to a PyTorch tensor, normalization using ImageNet mean and standard deviation, and random erasing with a probability of 0.1.
2. Load pre-trained models (AlexNet and ConvNexT) then freeze the parameters of the loaded models and create a new model by concatenating the output features of the two models and then adding a classifier layer.
3. Define a custom optimizer class that inherits from Adam with the modifications.
4. Define functions to get data loaders for training and validation then implement data loading and augmentation for the training set and the validation set.
5. Define the training loop using the optimizer and the loss “CrossEntropyLoss”.
6. Evaluate the model using the confusion matrix and the learning curve for the loss and the accuracy.

### Dataset

**Fig. 2:**
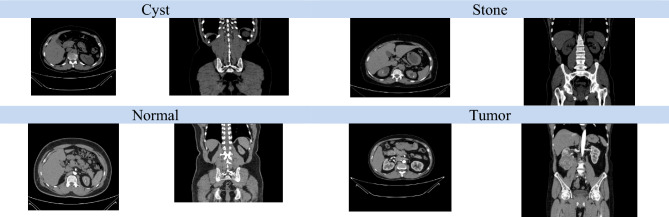
Sample images from the dataset.

The paper used the dataset that originated from various hospitals in Dhaka, Bangladesh, where patients had previously received diagnoses related to kidney tumors, cysts, normal conditions, or stone findings. The gathered data from the Picture Archiving and Communication System (PACS), incorporating both Coronal and Axial cuts from contrast and non-contrast studies covering the entire abdomen and urogram. Subsequently, patient information and metadata were excluded from the Dicom images, and the images were converted to a lossless jpg format. To ensure accuracy, each image finding underwent verification by both a radiologist and a medical technologist after the conversion process^14^. The dataset contains 12,446 unique data within it which the cyst contains 3709, normal 5077, stone 1377, and tumor 2283. As shown in Fig 2. The sample of the dataset used.

## Experiments and results

This study used the assembled and annotated 12,446 CT^14^ whole abdomen and urogram images that contained four classes Cyst, Normal, Stone, and Tumor as in Fig. 2. The paper divided the dataset into training and validation using augmentation to overcome the *overfitting* problem such as RandomHorizontalFlip, RandomVerticalFlip, CenterCrop, and Normalize the images. After augmentation training dataset be 19,450 instead of 9725 and the validation be 5442 instead of 2721.

**Fig. 3:**
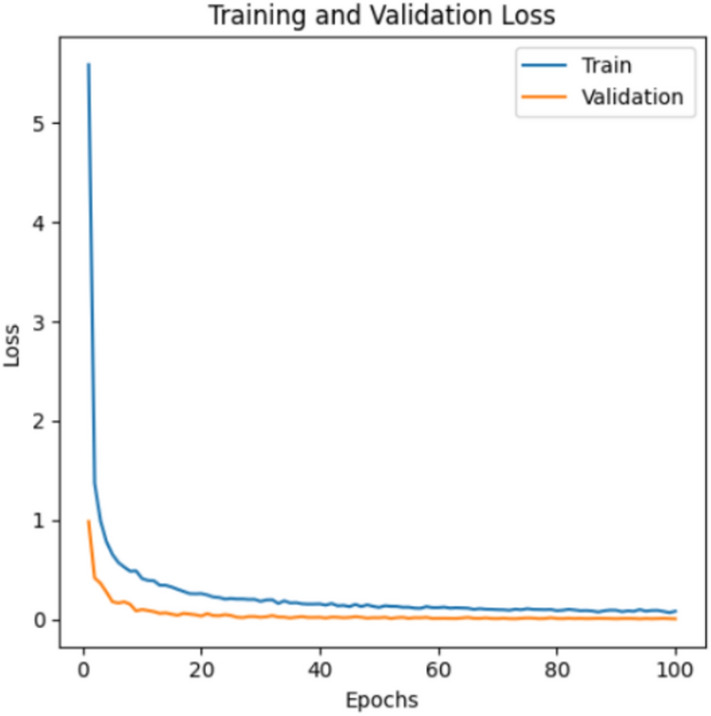
Training and Validation Loss.

**Fig. 4:**
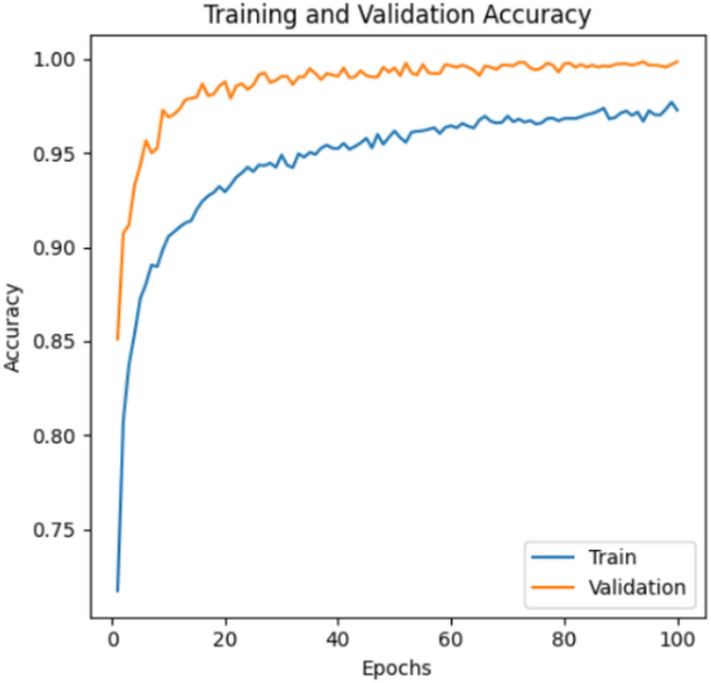
Training and Validation Accuracy.

**Fig. 5:**
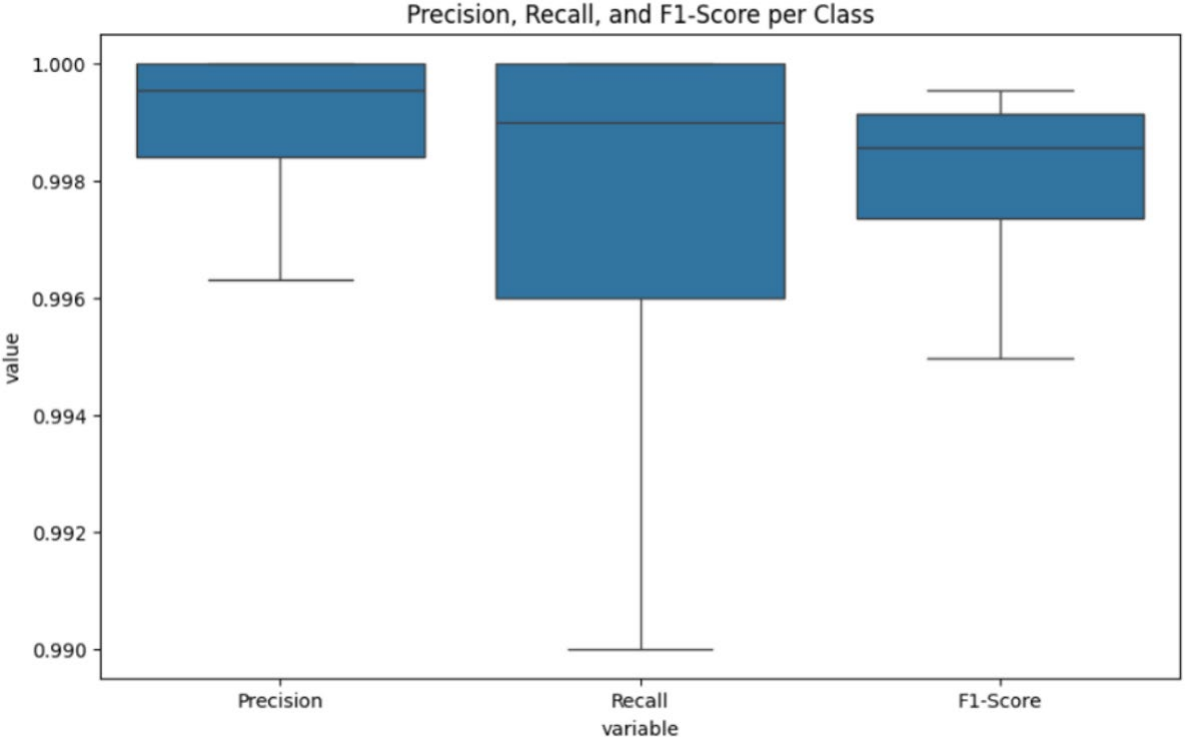
Precision, Recall, and F1-score for Alex-NeT + ConvNexT with custom-Adam optimizer.

The hyperparameter settings for the best model (Alex-NeT + ConvNexT with custom-Adam optimizer) are as follows: learning rate with 1e-4, Epochs = 100, loss = CrossEntropyLoss, Optimizer = custom-Adam and batch_size = 32. The paper trained the models using pytorch with a laptop with one GPU (2060 RTX). Figures 3 and 4 show the (training and validation loss) and (training and validation accuracy) respectively while Fig. 5 shows the Precision, Recall, and F1-score for the model.

### Performance evaluation methods

The evaluation of the eight models involves an analysis based on parameters such as accuracy in training, sensitivity (or recall), and precision (or positive predictive value - PPV). To calculate precision, and Recall, the paper utilizes true positive (TP), false positive (FP), true negative (TN), and false negative (FN) samples. Recall, also known as sensitivity, is determined by dividing the number of true positives by the sum of true positives and false negatives. In medical diagnosis, high recall is imperative for accurately identifying individuals with the disease, as overlooking the positive category can result in serious consequences like misdiagnosis and treatment delays. Precision (PPV) becomes crucial when assessing the proportion of predicted positive examples that are genuinely positive. Precision is calculated by dividing the number of true positives by the sum of true positives and false positives. In the realm of medical imaging, achieving high precision is highly desirable. The F1 score for all models is derived from the sensitivity and precision values. The provided formulas are applied to calculate accuracy, precision, sensitivity, and the F1 score^24^.

4 $${Percision}_{i}= \frac{{TP}_{i}}{{TP}_{i}+{FP}_{i}}$$

5 $${Recall}_{i}= \frac{{TP}_{i}}{{TP}_{i}+{FN}_{i}}$$

6 $${F-score}_{i}=\frac{2*{Percision}_{i}*{Recall}_{i}}{{Percision}_{i}+ {Recall}_{i}}$$

where, i=class of the kidney (Cyst or Normal or Stone or Tumor), TP= True Positive, FN= False Negative, TN=True Negative.

Table 2 shows the comparison between single vision models using the Adam optimizer and custom_Adam optimizer for the four classes of kidney diseases with some factors such as; accuracy, precision, recall, f-score, and the average for the four classes.

**Table 2:** Comparison between the individual models using the Adam and custom_Adam optimizer

Models	Optimizer	Class	Accuracy in %	Precision	Recall	F1 Score	The average for 4 classes
ViT	Adam	Cyst	**98.71**	0.9854	0.9941	0.9897	Average precision = **0.9923** Average recall = **0.9897**
		Normal		0.9955	0.9955	0.9955	
		Stone		0.9944	0.9852	0.9898	
		Tumor		0.9939	0.9840	0.9889	
	Custom_Adam	Cyst	98.57	0.9812	0.9926	0.9868	Average precision = 0.9918 Average recall = 0.9866
		Normal		0.9955	0.9928	0.9941	
		Stone		0.9964	0.9831	0.9897	
		Tumor		0.9939	0.9780	0.9859	
Alex-Net	Adam	Cyst	96.32	0.9517	0.9963	0.9735	Average precision = 0.9736 Average recall = 0.9626
		Normal		0.9846	0.9901	0.9874	
		Stone		0.9795	0.8815	0.9279	
		Tumor		0.9788	0.9823	0.9806	
	Custom_Adam	Cyst	96.91	0.9738	0.9703	0.9721	Average precision = 0.9772 Average recall = 0.9407
		Normal		0.9799	0.9917	0.9857	
		Stone		0.9770	0.8440	0.9053	
		Tumor		0.9780	0.9565	0.9672	
Swin	Adam	Cyst	96.44	0.9482	0.9938	0.9705	Average precision = 0.9795 Average recall = 0.9804
		Normal		0.9883	0.9765	0.9824	
		Stone		0.9921	0.9722	0.9821	
		Tumor		0.9894	0.9789	0.9841	
	Custom_Adam	Cyst	96.84	0.9471	0.9938	0.9700	Average precision = 0.9728 Average recall = 0.9797
		Normal		0.9874	0.9811	0.9842	
		Stone		0.9900	0.9629	0.9763	
		Tumor		0.9668	0.9812	0.9739	
ConvNexT	Adam	Cyst	96.88	0.9525	0.9926	0.9722	Average precision = 0.9831 Average recall = 0.9854
		Normal		0.9946	0.9737	0.9840	
		Stone		1.0	0.9777	0.9887	
		Tumor		0.9872	0.9978	0.9925	
	Custom_Adam	Cyst	96.62	0.9507	0.9915	0.9706	Average precision = 0.9816 Average Recall = 0.9833
		Normal		0.9937	0.9739	0.9837	
		Stone		1.0	0.9701	0.9848	
		Tumor		0.9821	0.9978	0.9898	

Significant values are in [bold].

The presented table summarizes the performance of various models, each employing different optimizers, in distinguishing between four classes: Cyst, Normal, Stone, and Tumor. Notably, Vision Transformer (ViT) models, both with Adam and Custom_Adam optimizers, consistently demonstrate robust accuracy, precision, and recall across the specified classes, showcasing their effectiveness in image classification tasks. Swin and ConvNexT models also exhibit commendable performance, with high accuracy and stable precision-recall metrics. Alex-Net models, while slightly lagging in accuracy, still demonstrate competitive results. The ViT model with Adam optimizer consistently demonstrates high accuracy across all classes, making it a strong contender. Precision and recall are often critical in medical imaging; the balance between the two might be preferred.

**Fig. 6:**
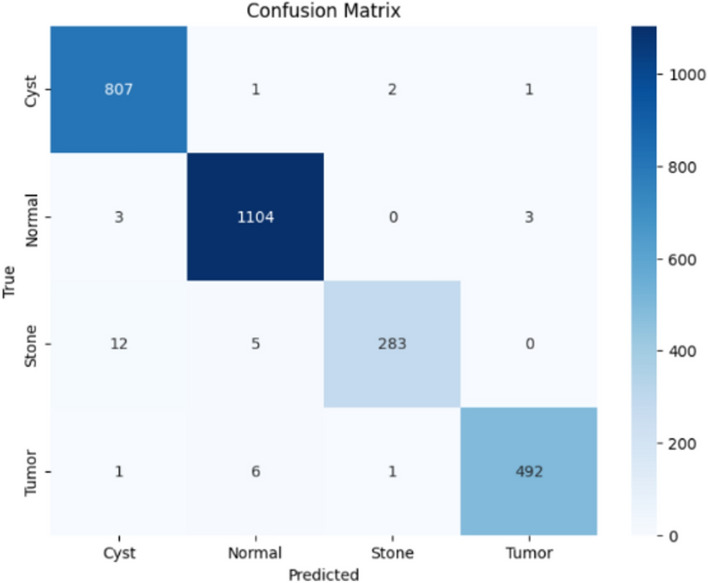
ViT with Adam optimizer model.

**Fig. 7:**
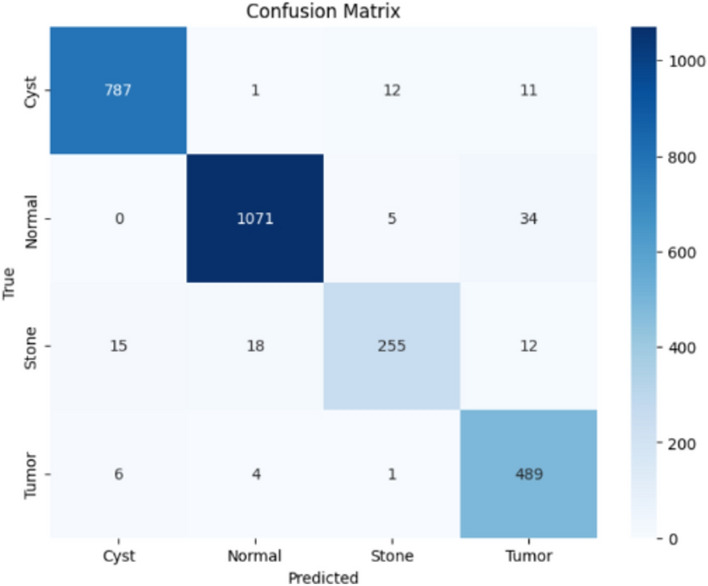
Alex-Net with custom_Adam optimizer.

**Fig. 8:**
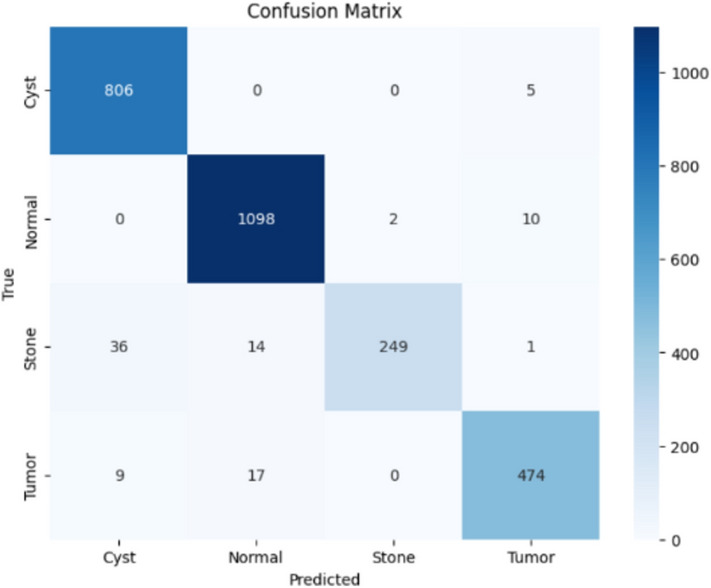
Swin with custom_Adam optimizer.

**Fig. 9:**
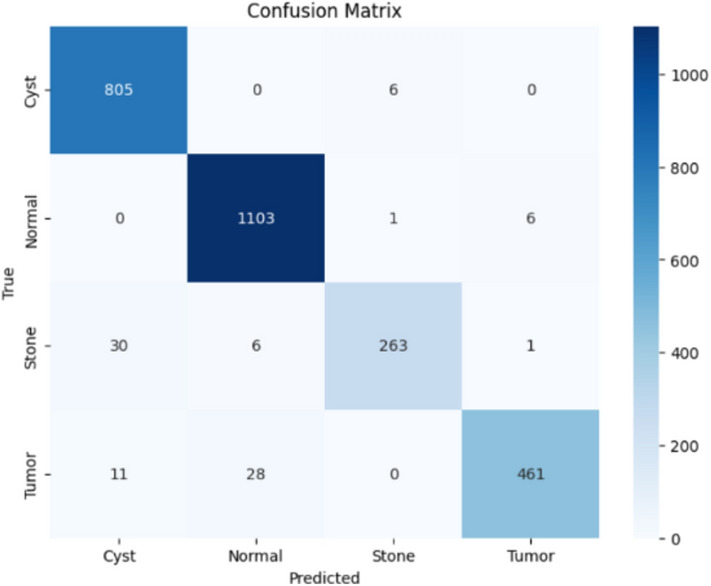
ConvNexT with Adam optimizer.

Here, the study presents the best confusion matrix for the four individual vision models utilizing Adam and custom_Adam, which demonstrates improved results in Figs. 6, 7, 8, and 9.

**Fig. 10:**
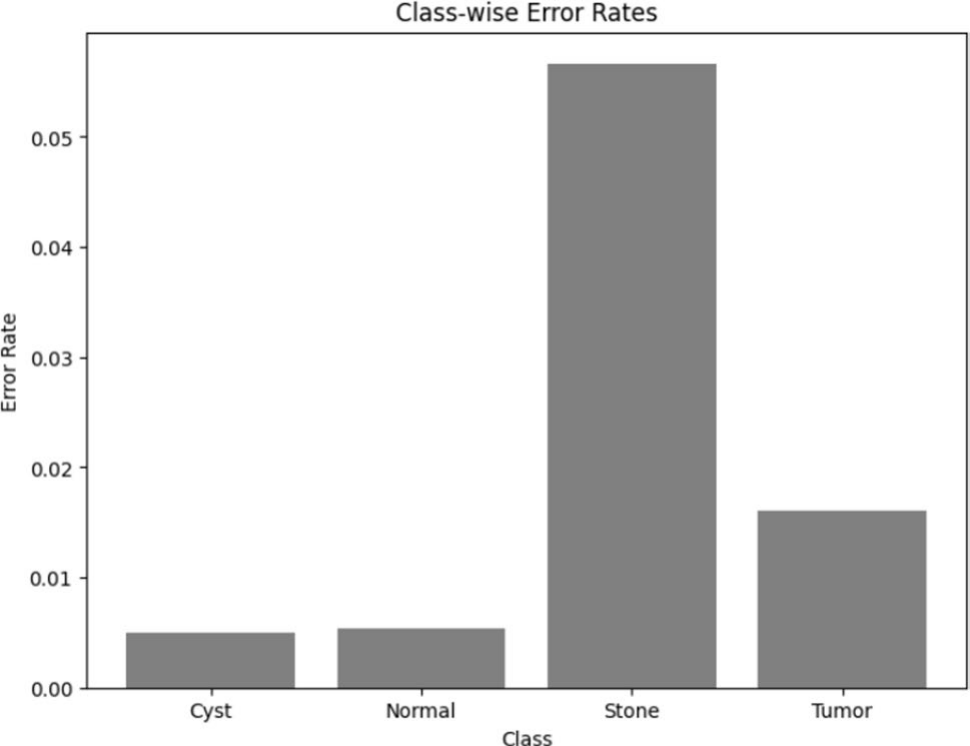
Class-wise error rate for ViT with Adam optimizer model.

**Fig. 11:**
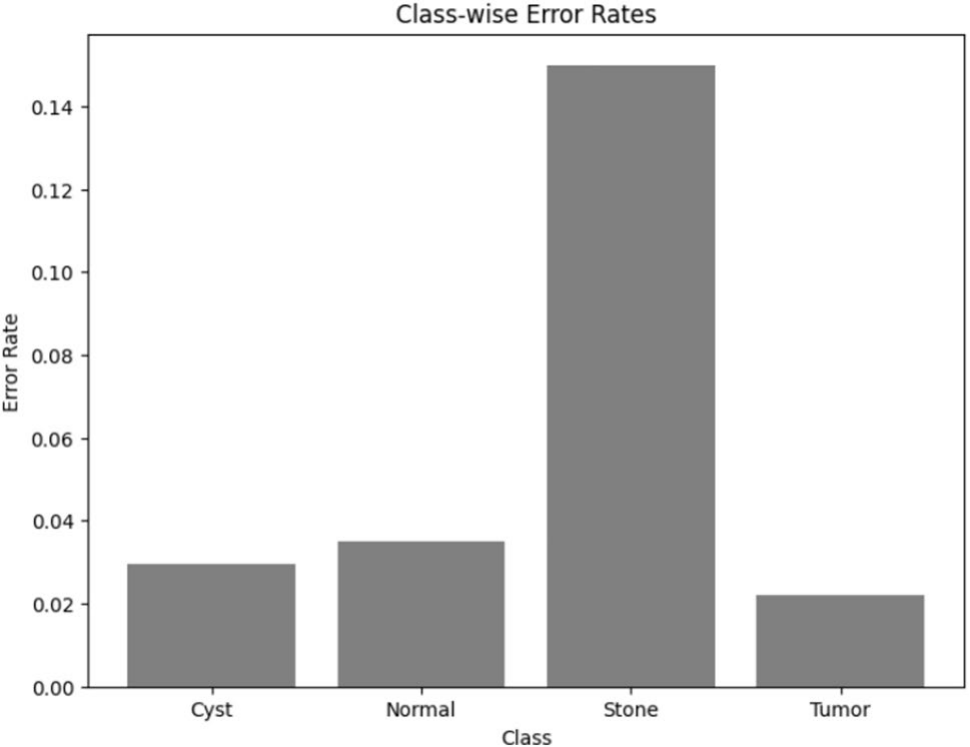
Class-wise error rate for Alex-Net with custom_Adam optimizer.

**Fig. 12:**
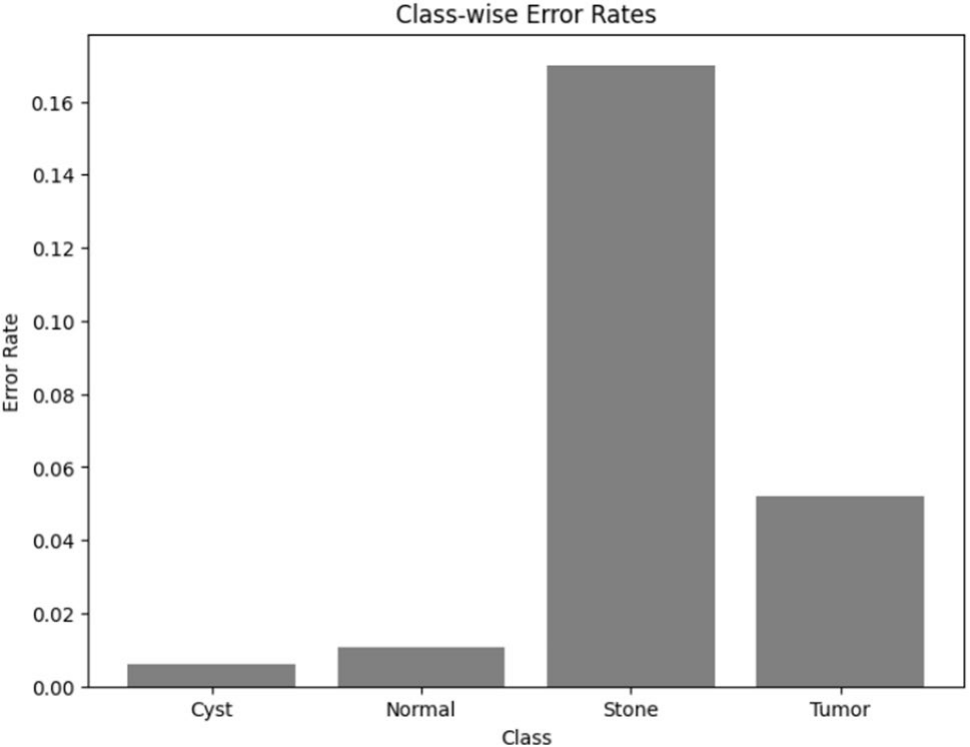
Class-wise error rate for Swin with custom_Adam optimizer.

**Fig. 13:**
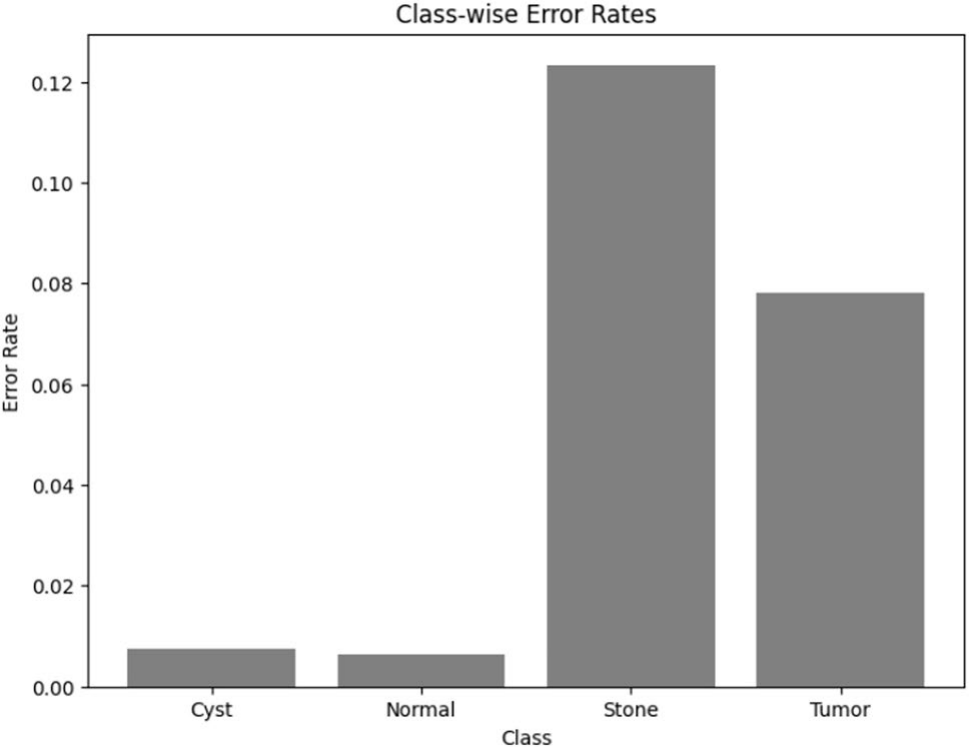
Class-wise error rate for ConvNexT with Adam optimizer.

Visualizing results using class-wise error rates is also essential for the evaluation of image classification models. This approach provides a detailed view of the model's performance across different categories. Unlike overall accuracy metrics, which aggregate performance across all classes, class-wise error rates highlight disparities in classification performance. It can offer a comprehensive understanding of model efficiency. Here is the class-wise error rate for the best four models the paper used in Figs. 10, 11, 12, and 13.

**Fig. 14:**
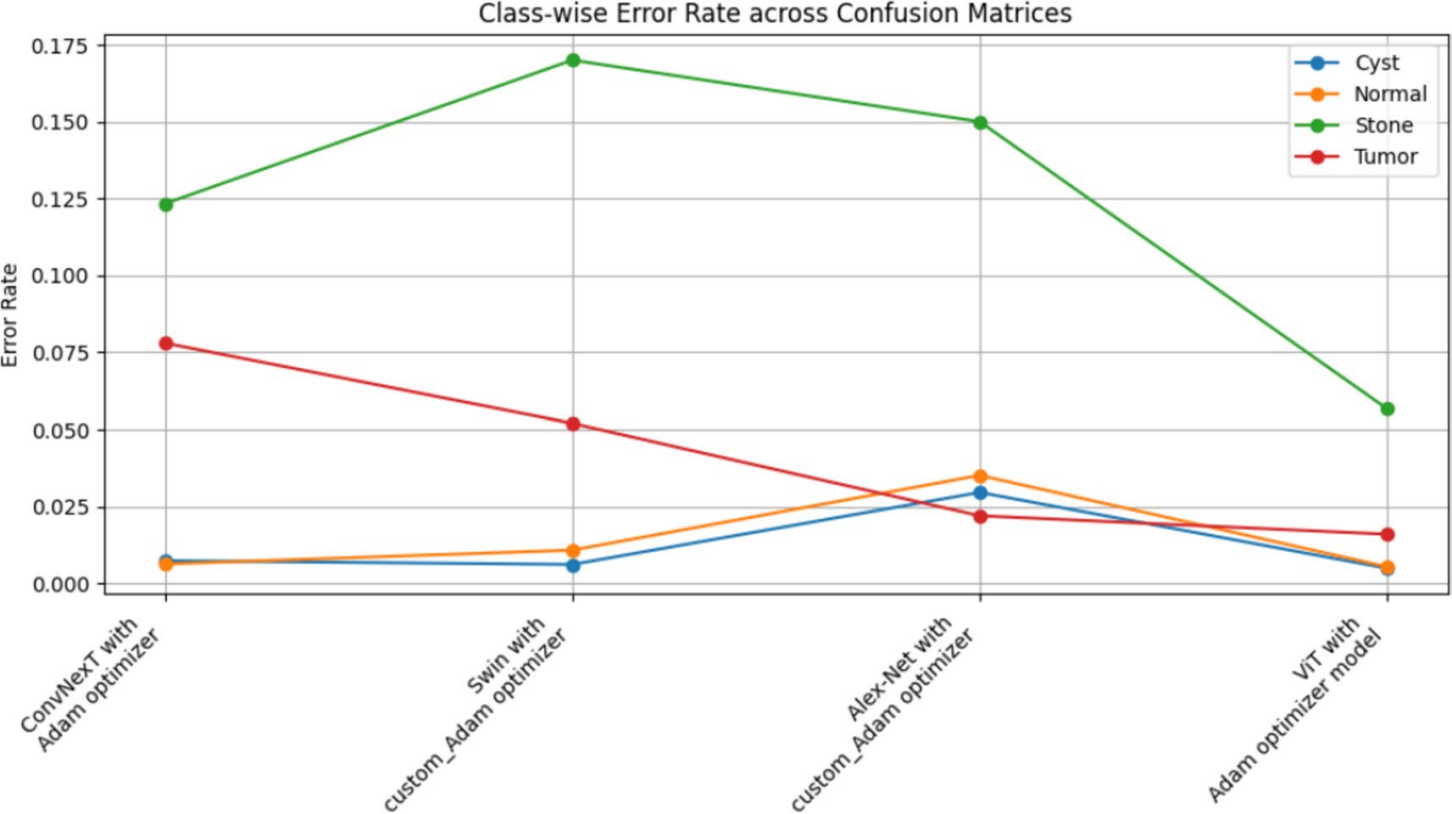
Class-wise error rate for the best four models.

The summarized comparison of the class-wise error rate between the best four models in Fig. 14

As in Fig. 14, all models consistently achieve near-perfect performance, with the second model (Swin with custom_Adam optimizer) achieving perfect classification. The error rates vary, with the third model (Alex-Net with custom_Adam optimizer) showing higher error rates, while the final model (ViT with Adam optimizer) shows the best performance. All models demonstrate strong performance with low error rates, with the second and fourth models showing the best performance. The best overall model appears to be the "ViT with Adam optimizer model", as it achieves the lowest error rates across most classes, demonstrating consistent and strong performance in classifying 'Cyst', 'Normal', 'Stone', and 'Tumor' samples.

Table 3 shows the comparison between concatenated vision models using Adam and custom_Adam optimizer for the four classes of kidney diseases with some factors such as; accuracy, precision, recall, f-score, and the average for the four classes.

**Table 3:** Comparison between the concatenated models using the Adam and custom_Adam optimizer.

Models	Optimizer	Class	Accuracy in %	Precision	Recall	F1 Score	The average for 4 classes
Swin + ConvNext	Adam	Cyst	99.12	0.9866	**1.0**	0.9932	Average precision = 0.9952 Average recall = 0.9932
		Normal		0.9982	0.9991	0.9987	
		Stone		0.9958	**1.0**	0.9979	
		Tumor		**1.0**	0.9739	0.9868	
	Custom_Adam	Cyst	98.75	0.983	1.0	0.9914	Average precision = 0.9949 Average recall = 0.9782
		Normal		0.9946	0.9991	0.9968	
		Stone		**1.0**	0.9429	0.9706	
		Tumor		**1.0**	0.9709	0.9853	
Alex-Net + Swin	Adam	Cyst	99.45	0.9818	0.9963	0.9890	Average Precision = 0.993 Average recall = 0.984
		**Normal**		0.9973	**1.0**	0.9986	
		Stone		0.9929	**0.9396**	0.9655	
		**Tumor**		**1.0**	**1.0**	**1.0**	
	Custom_Adam	Cyst	99.78	0.9951	0.9988	0.99695	Average precision = 0.9961 Average recall = 0.9986
		**Normal**		**1.0**	**1.0**	**1.0**	
		Stone		0.9933	0.9966	0.9950	
		**Tumor**		0.997	**1.0**	0.9985	
Alex-Net + ViT	Adam	Cyst	99.60	0.9832	**1.0**	0.9915	Average precision = 0.9951 Average recall = 0.9962
		**Normal**		**1.0**	0.9991	0.9996	
		Stone		**1.0**	0.9897	0.9948	
		**Tumor**		**1.0**	0.998	0.999	
	Custom_Adam	Cyst	99.74	0.9951	**1.0**	0.9975	Average precision = 0.9968 Average recall = **0.9995**
		Normal		0.9991	**1.0**	0.9995	
		Stone		0.9933	**1.0**	0.9966	
		Tumor		**1.0**	0.998	0.999	
**Alex-Net + ConvNexT**	Adam	**Cyst**	99.63	0.9926	**1.0**	0.996	average precision = 0.99735
		**Normal**		0.9973	**1.0**	0.9986	Average recall = 0.99755
		Stone		**1.0**	0.9966	0.9983	
		Tumor		**1.0**	**0**.9939	0.9968	
	**Custom_Adam**	**Cyst**	**99.85**	0.9963	**1.0**	0.9981	Average precision = **0.9989** Average recall = **0.9995**
		**Normal**		0.9991	**1.0**	0.9995	
		Stone		**1.0**	**1.0**	**1.0**	
		Tumor		**1.0**	0.998	0.999	

Significant values are in [bold].

Table 3 presents the effect of the concatenated features between the models. Alex-Net + ConvNexT with the custom_Adam stand out with the highest accuracy of 99.85%. On the other hand, the model with the lowest accuracy among those provided, Swin + ConvNexT with the custom_Adam optimizer with an accuracy of 98.75% has the lowest accuracy but its balanced precision and recall suggest effectiveness across various classes. But Alex-Net + ConvNext with the custom_Adam stands out with consistently high average precision (0.9989) and recall (0.9995) values, indicating robust performance across all classes. Among the provided models, the custom_Adam optimizer consistently outperforms the standard Adam optimizer in terms of accuracy, precision, recall, and F1-score in all concatenated models specifically the Alex-Net model with any Transformer model with the dynamic adjustment of the step size based on the norm of the gradient except of the Swin + ConvNexT model which give the less result with the custom_Adam and the Adam optimizer which may because the different architectures that make the model more complexity. Also if the gradient flow between Swin and ConvNexT is not well-aligned, the gradients might not propagate effectively during training, leading to convergence challenges.

**Fig. 15:**
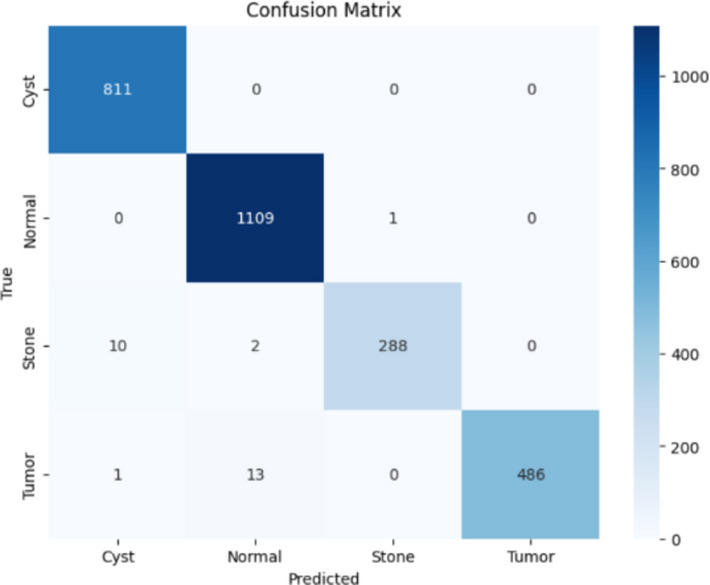
Swin + ConvNexT with Adam optimizer.

**Fig. 16:**
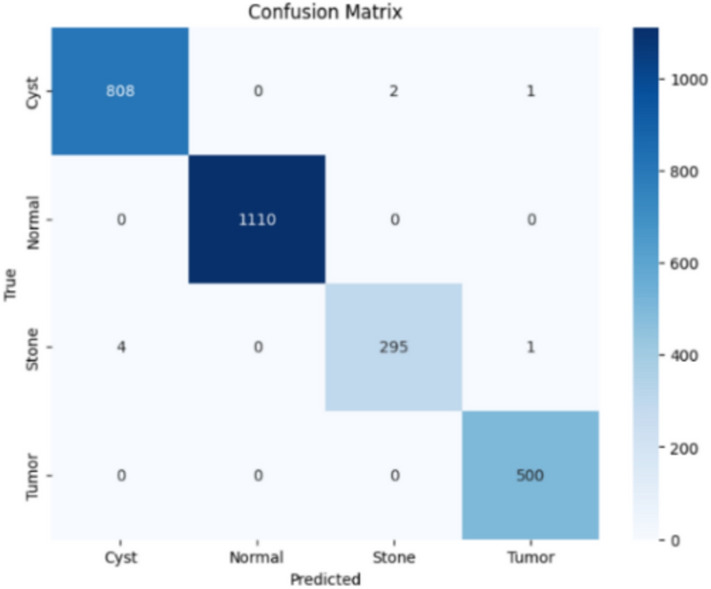
Alex-Net + Swin with custom_Adam optimizer.

**Fig. 17:**
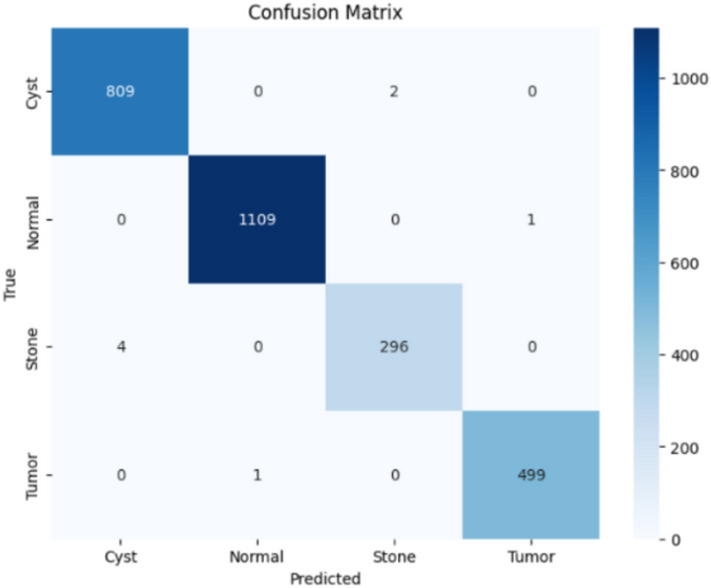
Alex-Net + ViT with custom_Adam optimizer.

**Fig. 18:**
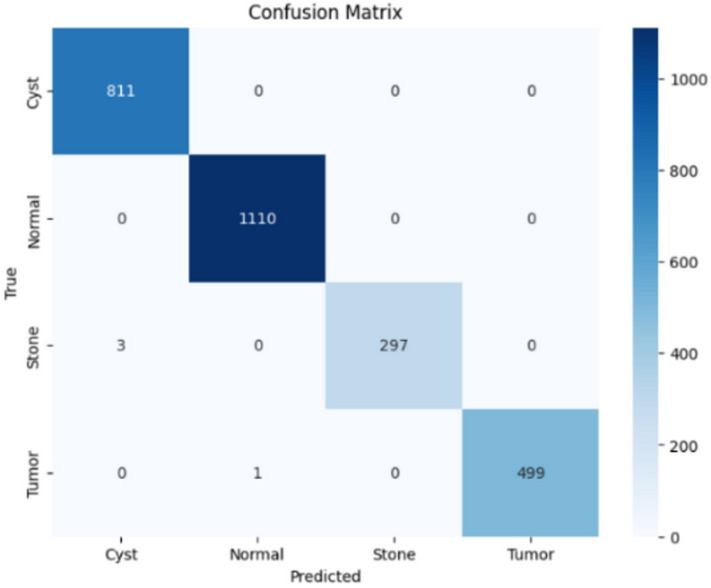
Alex-Net + ConvNexT with custom_Adam optimizer.

Here, the study presents the best confusion matrix for the four concatenated vision models utilizing Adam and custom_Adam, which demonstrates the best results in Figs. 15, 16, 17, and 18.

**Fig. 19:**
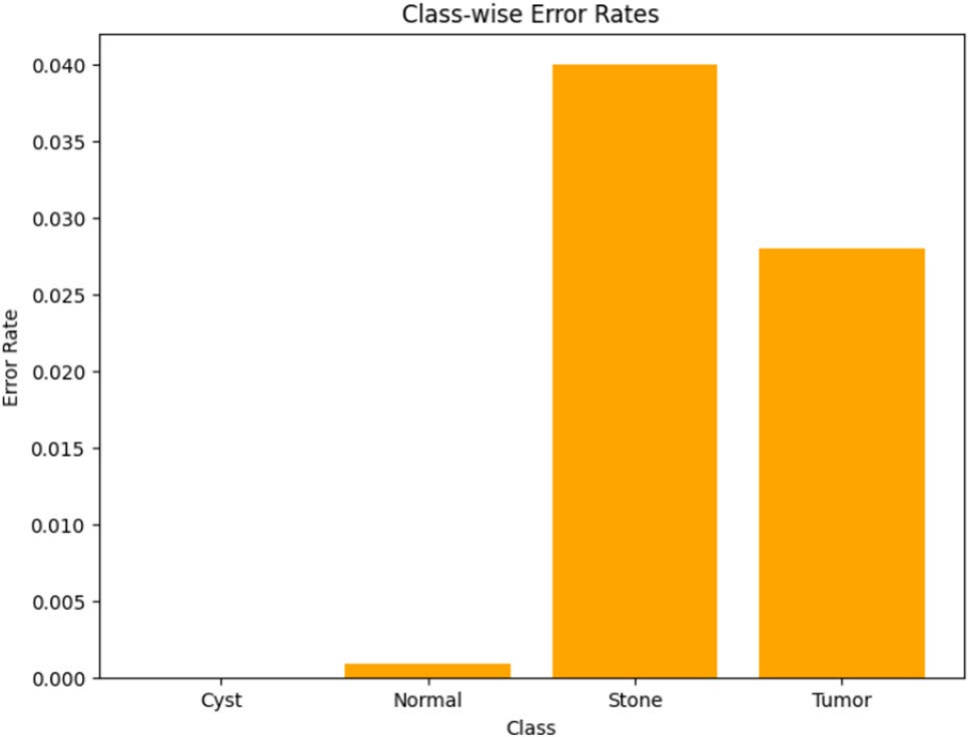
Class-wise error rate for Swin + ConvNexT with Adam optimizer.

**Fig. 20:**
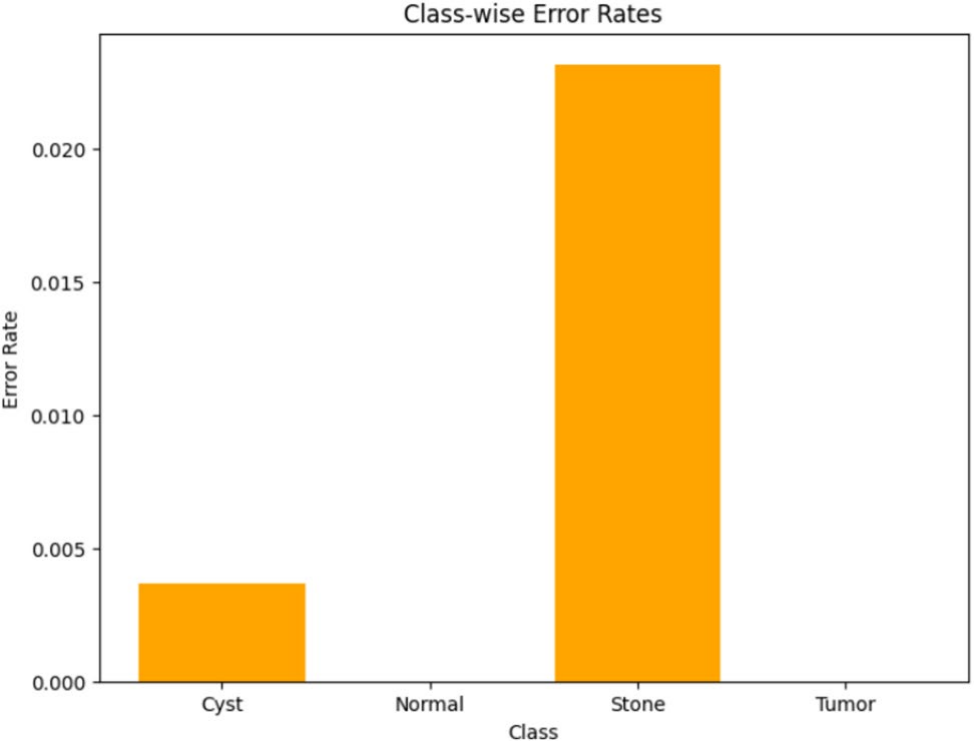
Class-wise error rate for Alex-Net + Swin with custom_Adam optimizer.

**Fig. 21:**
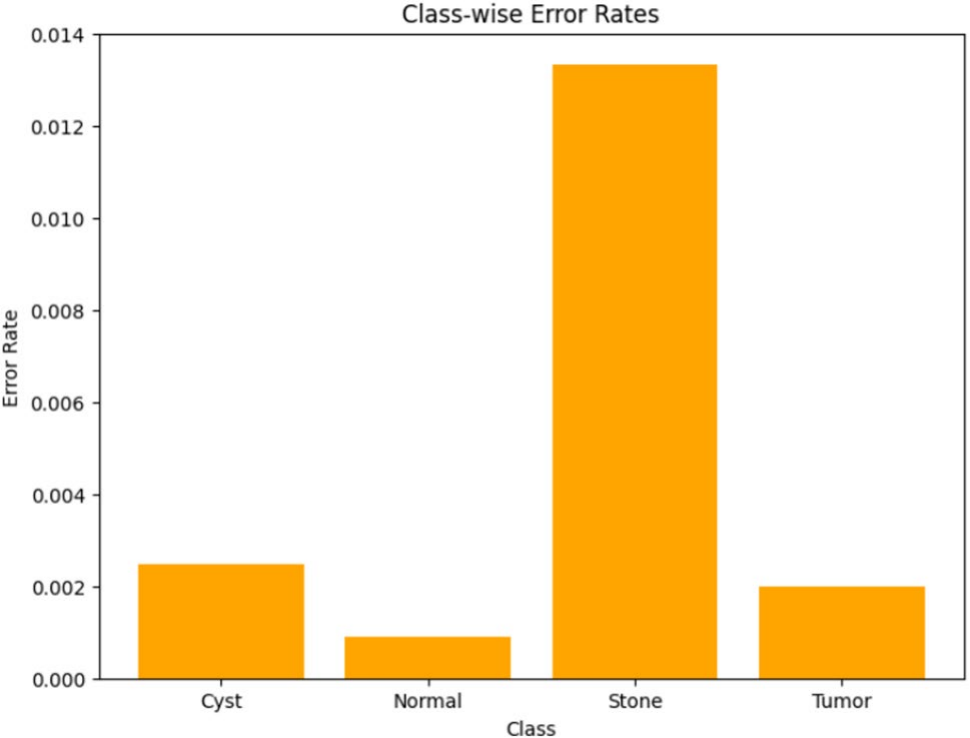
Class-wise error rate for Alex-Net + ViT with custom_Adam optimizer.

**Fig. 22:**
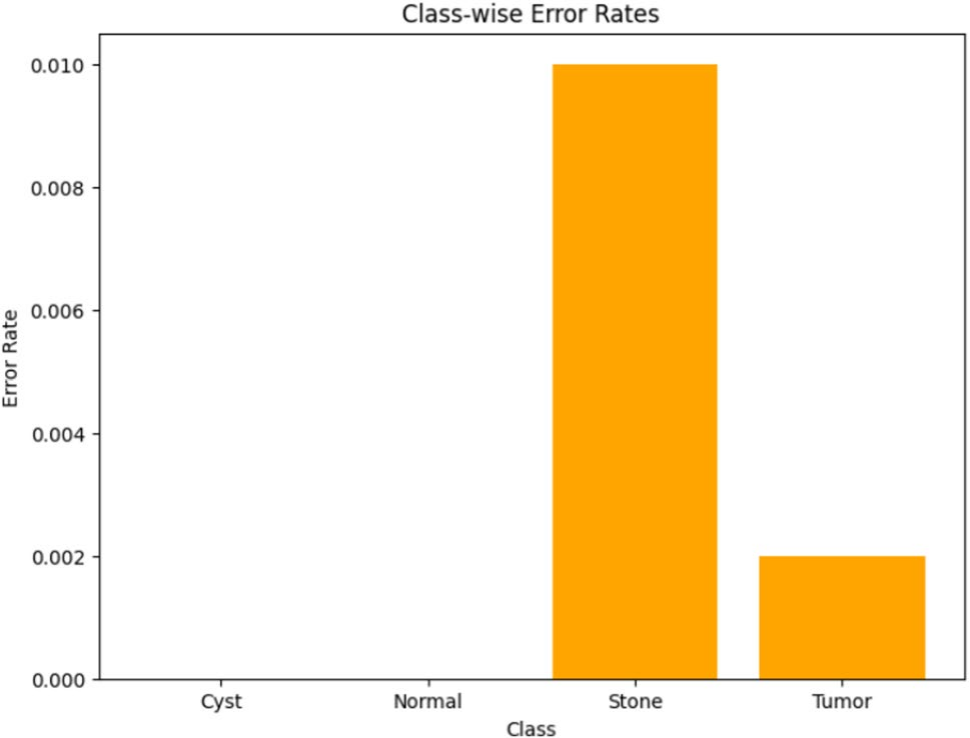
Class-wise error rate for Alex-Net + ConvNexT with custom_Adam optimizer.

Here is the class-wise error rate of the best concatenated models in Figs. 19, 20, 21, and 22.

**Fig. 23:**
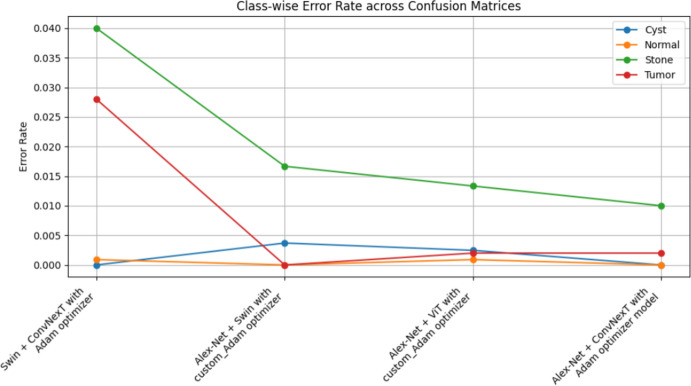
Class-wise error rate for the best four concatenated models.

The summarized comparison for the class-wise error rate between the best four concatenated models in Fig. 23

As in Fig. 23, all models perform well in classifying 'Cyst' samples, with the final model (Alex-Net + ConvNexT with Adam Optimizer) showing perfect performance. Also, all models consistently achieve near-perfect or perfect performance in classifying 'Normal' samples, with multiple models achieving perfect performance. The error rates vary slightly, but all models generally perform well, with the second model (Alex-Net + Swin with custom_Adam Optimizer) showing the best improvement. All models demonstrate strong performance, with very low error rates across the board. The second model (Alex-Net + Swin with custom_Adam Optimizer) and the final model (Alex-Net + ConvNexT with Adam Optimizer) show perfect or near-perfect performance. The best overall model appears to be the "Alex-Net + ConvNexT with Adam Optimizer Model", as it achieves perfect classification in the 'Cyst' and 'Normal' classes, very low error rates in the 'Stone' class, and almost perfect performance in the 'Tumor' class. This model consistently demonstrates strong performance across all classes, making it the most reliable and effective model in this comparison.

### No. parameters of different models

**Fig. 24:**
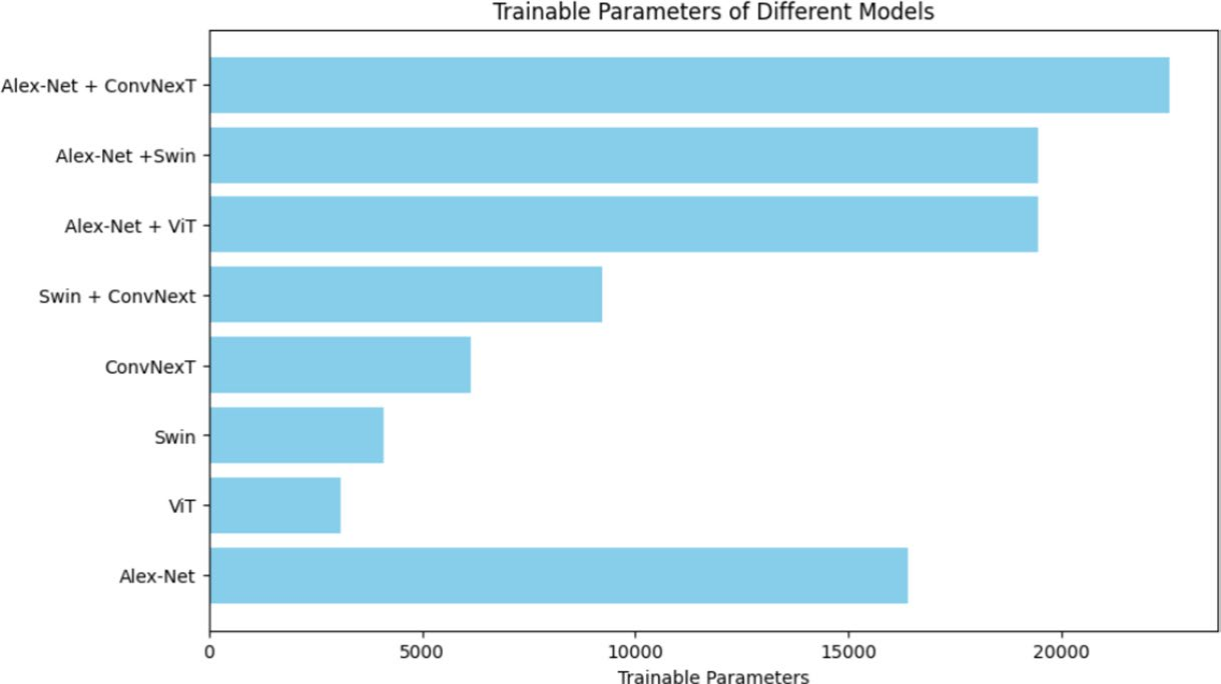
Trainable parameters for the models used in the paper.

One essential feature that greatly affects a neural network model's capacity, efficiency, and flexibility is the number of parameters. Deep learning models consist of several layers, each of which has weights and biases that add to the total number of parameters. Greater representational capacity is often possessed by larger, more parameterized models, which allows them to learn complex characteristics and relationships in data. Conversely, more compact models with fewer parameters could be less prone to ***overfitting*** and more computationally efficient, which makes them appropriate for jobs requiring sparse data. As shown in Table 4, the total number of parameters and trainable parameters for the single models and the concatenated models used in this paper. It's generally more meaningful to focus on "Trainable parameters" rather than "Total number of parameters." because not all parameters in a model may be trainable, as some might be fixed or non-trainable. As in Table 4, the model with the least parameters is Swin, and the model with the most parameters is Alex-Net + ConvNexT. Larger parameter counts are often associated with better model accuracy, so the progression from the model with the least parameters to the most parameters could represent an increase in model capacity and, potentially, accuracy as in Fig. 24.

**Table 4:** No. of parameters of the single and concatenated models.

Model-Name	Total number of parameters	Trainable parameters
ViT	85,801,732	3076
Alex-Net	**57,020,228**	16,388
Swin	86,909,948	**4100**
ConvNexT	196,236,484	6148
Swin + ConvNext	223,755,838	9220
Alex-Net + Swin	84,542,654	19,460
Alex-Net + ViT	142,821,956	19,460
**Alex-Net + ConvNexT**	**253,253,636**	**22,532**

Significant values are in [bold].

### Time evaluation

**Fig. 25:**
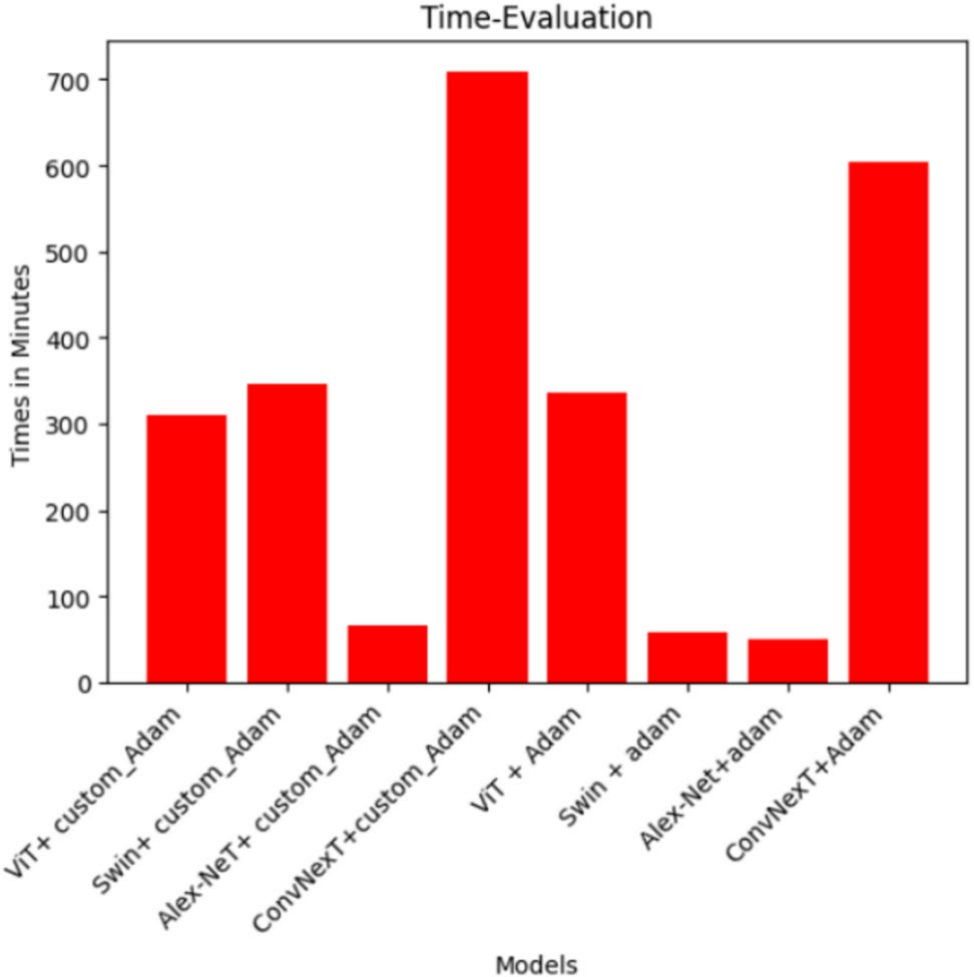
Time evaluation for training the eight single models.

For each of the 8 tested single models, the study compared the time taken for training for each model to get the less time that was taken. As shown in Fig. 25, Alex-Net with Adam optimizer was the fastest in training as it took the least training time (50 minutes) with an accuracy of 96.32 followed by Swin with Adam optimizer which took 59 minutes with an accuracy of 96.44 then Alex-Net+ custom_Adam that took 66 minutes with accuracy 96.91 while the best one which is ViT with Adam optimizer took 5 hours approximately with accuracy 98.71 while the ConvNexT with custom_Adam optimizer took the longest time with around 10 hours with an accuracy of 96.62.

**Fig. 26:**
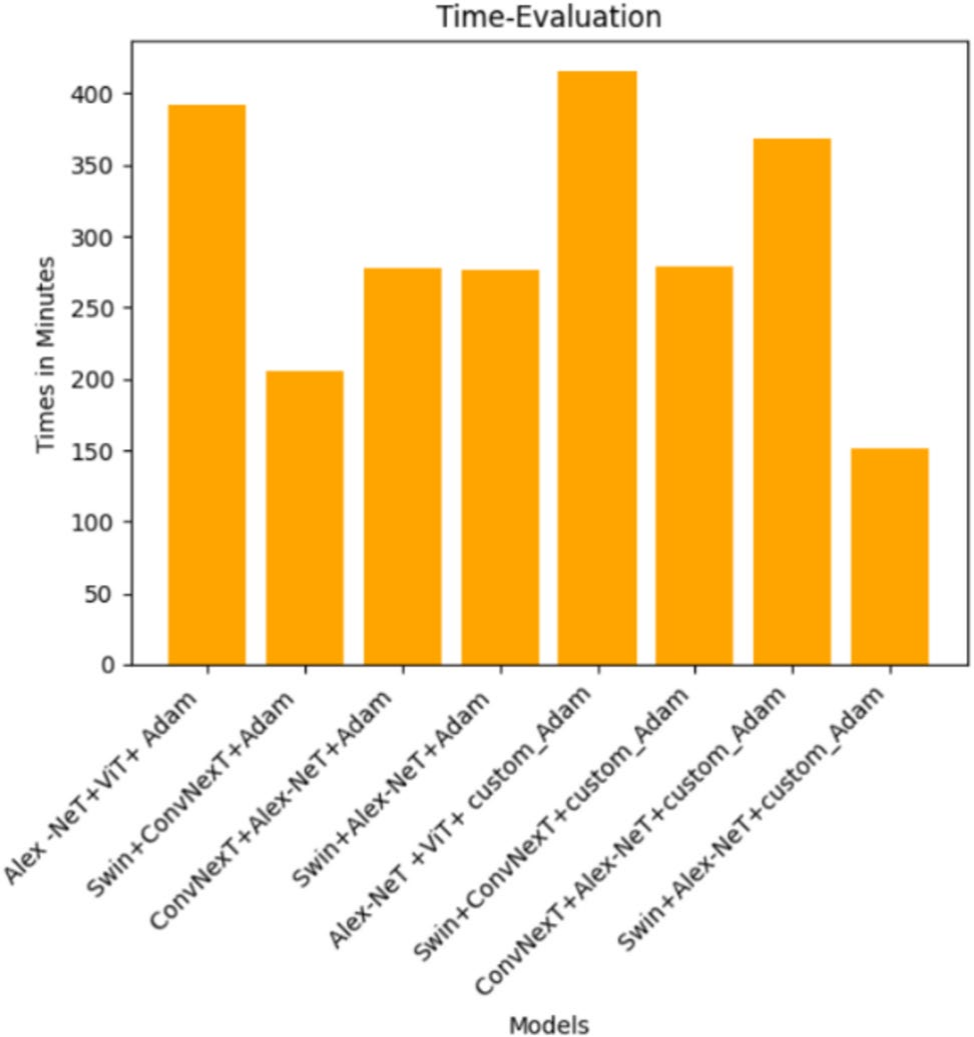
Time evaluation for training the eight concatenated models.

As shown in Fig. 26, the concatenated models for each of the 8 tested concatenated models, the paper also compared the time taken for training for each model to get the less time that taken., Swin + Alex-Net with custom_Adam optimizer was the fastest in training as it took the least training time (2 h and 30 min) with an accuracy of 99.78 followed by Swin + ConvNexT with Adam optimizer which took around 3 h and a half with an accuracy of 99.12 while ConvNexT + Alex-Net, Swin + Alex-Net with Adam optimizer and Swin + ConvNexT with custom_Adam optimizer took the same time around 4 h and a half with accuracies 99.63, 99.45 and 98.75 respectively. The best one which is ConvNexT + Alex-Net with cuatom_Adam optimizer took 6 h approximately with an accuracy of 99.85. While the Alex-Net + ViT with custom_Adam optimizer took the longest time around 7 h with an accuracy of 99.74.

## Conclusions

This study explored the impact of feature concatenation and optimizer selection on neural network performance. The experimental results reveal that concatenating features, such as Alex-Net + ConvNexT, in combination with the custom_Adam optimizer, achieved an impressive accuracy of 99.85%. This highlights the benefits of integrating diverse model architectures and optimizing strategies to capture complex patterns and correlations in data. The custom_Adam optimizer demonstrated superior performance compared to the standard Adam optimizer across all concatenated models, excelling in accuracy, precision, recall, and F1-score. Particularly notable was its effect when paired with Transformer models, where dynamic step size adjustments based on gradient norms contributed to consistently high average recall and accuracy. The trade-off between model capacity and efficiency was evident, with the Swin model, despite its fewer parameters, performing competitively. This underscores its utility in scenarios where computational efficiency and reduced overfitting are critical. While larger models like Alex-Net + ConvNexT exhibited higher accuracy, the Swin + Alex-Net combination offered a balanced approach with a training duration of 2 h and 30 min and an accuracy of 99.78%. Conversely, the Alex-Net + ViT configuration, though achieving 99.74% accuracy, required the longest training time of approximately 7 h.

## Data Availability

The data that support the findings of this study are available from https://www.kaggle.com/datasets/nazmul0087/ct-kidney-dataset-normal-cyst-tumor-and-stone.
